# Characterization of bacterial nanocellulose cultivated on polyethylene terephthalate (PET) monomers via raman and fourier transform infrared spectroscopy

**DOI:** 10.1038/s41598-026-46886-z

**Published:** 2026-04-22

**Authors:** Ronja Eriksson, Iqra Mariam, Kerstin Ramser, Alok Patel

**Affiliations:** 1 Department of Engineering Sciences and Mathematics, Luleå University of Technology, Luleå, 971 87 Sweden; 2 Biochemical Process Engineering, Division of Chemical Engineering, Department of Civil, Environmental, and Natural Resources Engineering, Luleå University of Technology, Luleå, SE-971 87 Sweden

**Keywords:** Bacterial nanocellulose, Carbon sources, Ethylene glycol (EG), Disodium terephthalate (Na_2_-TPA), Raman spectroscopy, Fourier Transform Infrared (FTIR) spectroscopy, Chemistry, Materials science, Nanoscience and technology

## Abstract

The potential of *Komagataeibacter (K.) sucrofermentans* to valorize polyethylene terephthalate (PET) monomers - ethylene glycol (EG) & disodium terephthalate (Na_2_-TPA) - and glucose into bacterial nanocellulose (BNC) membranes was investigated using Raman and Fourier transform infrared (FT-IR) spectroscopy. Gravimetric analysis indicated higher BNC yields for *K. sucrofermentans* nurtured with EG (2.54 g L^− 1^) compared to pure glucose (2.00 g L^− 1^) and TPA (1.54 g L^− 1^). Scanning Electron Microscopy (SEM) images showed that BNC derived from glucose had a dense interconnected fibrillar matrix with uniform porosity, whereas EG and TPA displayed a network structure with less uniform fiber packing. The Segal approach from X-ray diffraction (XRD) showed a lower crystallinity for EG (CrI 62.3%) and substantially lower for TPA (CrI of 18%) than for the commonly known values of glucose (CrI around 70%). Raman spectra of BNC showed that bands between 1050 cm^− 1^ and 1150 cm^− 1^ were shifted towards lower wavenumbers compared to AVICEL cellulose, indicating an increased strain on the glycosidic linkages. The Raman spectrum of BNC with TPA as a feedstock showed additional bands at 629, 861, 1125, and 1425 cm^− 1^ indicating that not all TPA was valorized. FT-IR spectra of the BNC with EG and TPA as feedstocks showed minor transmission and bands at 447, 560, 687, 730, 862, plus between 1500 and 1600 cm^− 1^ that are typically assigned to PET monomers. The absorption ratio A ~ 1430/ A ~ 897, a measure of crystallinity, gave the values glucose = 1.02, TPA = 0.63, EG = 0.53 showing that glucose as a feedstock produced the highest BNC crystallinity. In the case of glucose and EG, well-structured cellulose membranes were produced, whereas for TPA a composite membrane was produced, paving the way to use PET monomers as a feedstock for biodegradable BNC production.

**Supplementary Information:**

The online version contains supplementary material available at 10.1038/s41598-026-46886-z.

## Introduction

Cellulose is an integral component of the cell wall of plants, algae and fungi, making it the most abundant biopolymer globally^1^. Cellulose has a wide range of applications including cotton, linen, and rayon for clothing, paper goods, cellulose acetate for packaging films, and construction wood. Because it is stable, non-toxic, and renewable, it is also used to produce industrial sugars, medicines, energy drinks and as an excipient in the formulation of solid oral doses^2,3^. Cellulose is traditionally produced from plant matter sourced from deforestation or monocultures causing environmental concerns^4^. As a result, much attention has been directed toward new ways of synthesizing cellulose using new sources such as microalgae and bacterial systems. Bacterial species belonging to the major *Acetobacteraceae* family, such as *Gluconacetobacter*, can efficiently produce nanostructured cellulose during microbial fermentation. Other bacterial genera that can produce bacterial nanocellulose (BNC) include *Agrobacterium*,* Pseudomonas*,* Sarcina*,* Burkholderia*,* Aerobacter*, and *Komagataeibacter* (formerly *Gluconacetobacter*)^5,6^.

The composition of BNC is similar to plant-derived cellulose, i.e. (C_6_H_10_O_5_)_n_, which is a homopolymer chain of glucose monomers connected with β-(1 → 4) glycosidic linkages^7^. These polymers have high tensile strength attributed to the multiple hydrogen bonds between the OH groups of glucose molecules and the oxygen atoms on neighboring or on the same chain. This results in a tight interaction between cellulose fibrils imparting crystalline structures of the BNC^8^. Higher-order BNC is synthesized during fermentation, which subsequently aggregates to form ribbon-shaped microfibrils, which are among the thinnest naturally occurring fibers with a width of less than 100 nm^9^. BNC is a highly useful biopolymer with superior mechanical and physicochemical properties compared to plant cellulose. The large surface area per unit of nanofibres, along with the hydrophilic characteristics of BNC, delivers high water absorption and retention capacity with improved adherence^10^. Along with better mechanical properties, these BNCs have high biodegradability and can be easily purified since their production is free of hemicellulose, pectin, and lignin, which are otherwise components of plant cell walls^10^. BNC has significant potential for various applications in industries such as food, papermaking, acoustic diaphragms, cosmetics, and biomedicine^11,12^. Additionally, compared to other celluloses, it has more surface hydroxyl and ether groups, which enhances its ability to adsorb metal ions^11^.

Although the BNC has several advantages over conventional cellulose and a broad range of applications, lower cellulose productivity and high input cost for microbial fermentation render the process economically unfeasible. The major cost is associated with carbon sources used for bacterial cultivation. Thus, alternative carbon sources such as sucrose, molasses, sugar cane, and rotten fruit have been utilized for BNC production^13^. However, shifting to sustainable or alternative carbon substrates results in BNC yields with varying structural and mechanical attributes. Apart from conventional sugars, several bacterial species are reported to use organic compounds derived from various waste streams, such as acetic acid, as energy sources^14^. Polyethylene terephthalate (PET) is one of the major recalcitrant pollutants and causes an alarming pollution problem worldwide. Several physical and chemical methods are employed to depolymerize PET into its constituent monomers, i.e. ethylene glycol (EG) and terephthalate (TPA). These plastic monomers can be further utilized by microbes including bacteria and algae to produce value-added products including polyhydroxyalkanoates (PHA), biofuels, and cellulose^15,16^. Using plastic monomers as an alternative feedstock would be most beneficial since one of the major global concerns, microplastic pollution, can be addressed by valorizing polymeric micro/nanoparticles into valuable resources for food packaging or for wound dressing. The use of commercial plastic monomers as a feedstock for bacterial species to produce biodegradable nanocellulose may pave the way to use microplastics from waste, which to the best of our knowledge has so far not been achieved yet^17^.

Raman spectroscopy is a label-free, nondestructive, and noncontact laser based spectroscopic method based on inelastic light scattering from molecular vibrations. Raman spectroscopy can give structural information and has, since the 1970 s, been used to investigate cellulose^18,19^. In 1987, it was shown that several vibrational modes could contribute to a single Raman band due to the large number of molecular degrees of freedom present in cellulose^20^. In the last decades, Raman spectroscopic studies of cellulose have increased, and several methods have been developed to characterize the different properties of cellulose, such as cellulose I, cellulose II, and amorphous cellulose^21^. Raman spectroscopy has been used to estimate the degree of crystallinity^22–24^, to detect and quantify cellulose II in a sample^25^, and to estimate the chain conformational disorder and water accessibility^26^. Raman spectroscopy has also been used to investigate the application of cellulose for (i) food packaging^27^, (ii) wound dressing^28,29^, and (iii) for seawater filtration as a component of cellulose/graphene oxide membranes^30^. Raman spectroscopy has further been used to study the differences between cellulose from fruit and bacteria^31^, and to investigate how the physical properties of bacterial cellulose are influenced by the presence of pectin and hemicelluloses in the bacterial growth environment^32^.

A complementary method to Raman spectroscopy is Fourier transform infrared (FT-IR) spectroscopy. The selection rule of Raman spectroscopy, a change in polarization, makes it particularly useful for symmetric molecules, whereas the selection rule for FT-IR, a change in dipole moment, makes it excellent to study asymmetric molecules. The benefit of Raman spectroscopy is that there is no spectral interference of water in the most interesting regions, however, it is often influenced and even hampered by fluorescence due to its wavelength range between 380 and 1100 nm. This is not an issue for FT-IR that operates in the infrared region; however, water signatures may disturb other spectral signatures. FT-IR spectroscopy has extensively been used to study BNC derived from different bacterial strains or food sources in regard of productivity and structural properties using a variety of feedstocks^33–35^.

In this study, we aimed to assess the potential of *K. sucrofermentans* (DSM 15973) to valorize PET monomers, i.e. EG and TPA, into biodegradable bacterial nanocellulose. As screening tools Raman and FT-IR spectroscopy were used to investigate potential biomolecular and structural differences between BNC cultured with EG or TPA. These results were compared with those of BNC cultured with pure glucose and with a commercially available cellulose (Avicel). The cellulose structure was confirmed and compared to Scanning Electron Microscopy (SEM) images and X-Ray Diffraction (XRD).

## Results and discussion

### Valorization of PET monomers to BNC

**Fig. 1:**
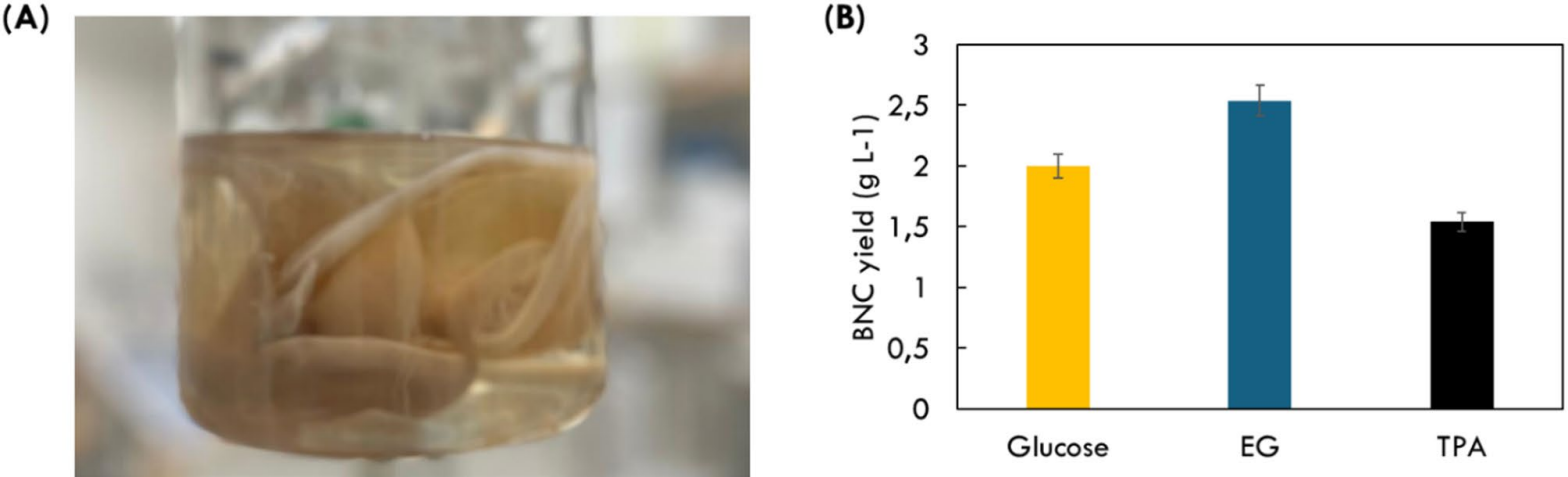
(**A**): Bacterial nanocellulose (BNC) synthesized by K. sucrofermentans (**B**) Estimated BNC yields (gL^− 1^) after lyophilization for K. sucrofermentans cultivated in the presence of 20 gL^− 1^ of glucose, ethylene glycol (EG) and terephthalic acid (TPA).

BNC production by bacteria is highly dependent on several cultivation parameters such as carbon source, pH, aeration, nitrogen source, and agitation^36^. The most vital component responsible for both yield and process cost is the carbon source. Here, *K. sucrofermentans* was cultivated for BNC production in the presence of HS supplemented with 20 gL^− 1^ of ethylene glycol or disodium terephthalate, respectively, see Fig. 1A. In the presence of glucose, the BNC yield obtained was 2.0 g L^− 1^, while in the presence of EG, the BNC yield was relatively higher i.e., 2.54 gL^− 1^. *K. xylinus* cultivated in the presence of disodium TPA resulted in a relatively low BNC yield (1.54 gL^− 1^), see Fig. 1B. Esmail et al., performed a similar study using the *K. xylinus* strains DSM 2004 and DSM 46,604^37^; where the former was found to have relatively lower BNC yields i.e., 0.64 & 0.81 g L^− 1^ in the presence of EG & TPA as compared to *K. sucrofermentans* used in this study. The higher productivity of BNC obtained for *K. sucrofermentans* in the presence of EG used here suggests efficient metabolic machinery.

### Scanning electron microscopy (SEM) analysis

**Fig. 2:**
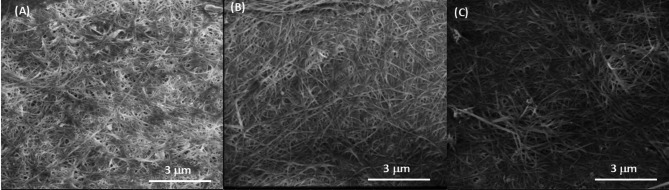
SEM image of BNC pellicle produced by *K. sucrofermentans* cultivated in (**A**) Glucose (**B**) EG & (**C**) TPA.

The morphology of the BNC pellicles, synthesized from glucose, ethylene glycol (EG), and terephthalic acid (TPA), was investigated using scanning electron microscopy (SEM). To ensure a direct and unambiguous comparison, all samples were analyzed under identical, optimized conditions: an accelerating voltage of 3.00 kV, a beam current of 6.3 pA, and using the Everhart-Thornley Detector (ETD) for topographical contrast. The samples were imaged directly as sheets at a high magnification of approximately 15,000x, see Fig. 2.

All samples exhibited the characteristic three-dimensional, nanofibrous network typical of BNC. However, distinct differences in network density and architecture were observed contingent upon the carbon source. The BNC derived from glucose (Fig. 2A) presented a dense, highly interconnected fibrillar matrix with uniform porosity, a morphology indicative of robust and homogeneous biosynthesis^17^. In contrast, the BNC produced from the alternative carbon sources, EG and TPA (Fig. 2B & C), displayed a visibly more porous and open network structure with less uniform fiber packing, with EG being closer to the glucose based BNC structure. The less dense, more open architecture observed in the EG- and TPA-derived BNC suggests that the metabolic processing of these non-conventional substrates impacts not only the molecular arrangement but also the ultimate nano-fibrillar assembly of the cellulose.

### X-Ray Diffraction analysis of BNC

**Fig. 3:**
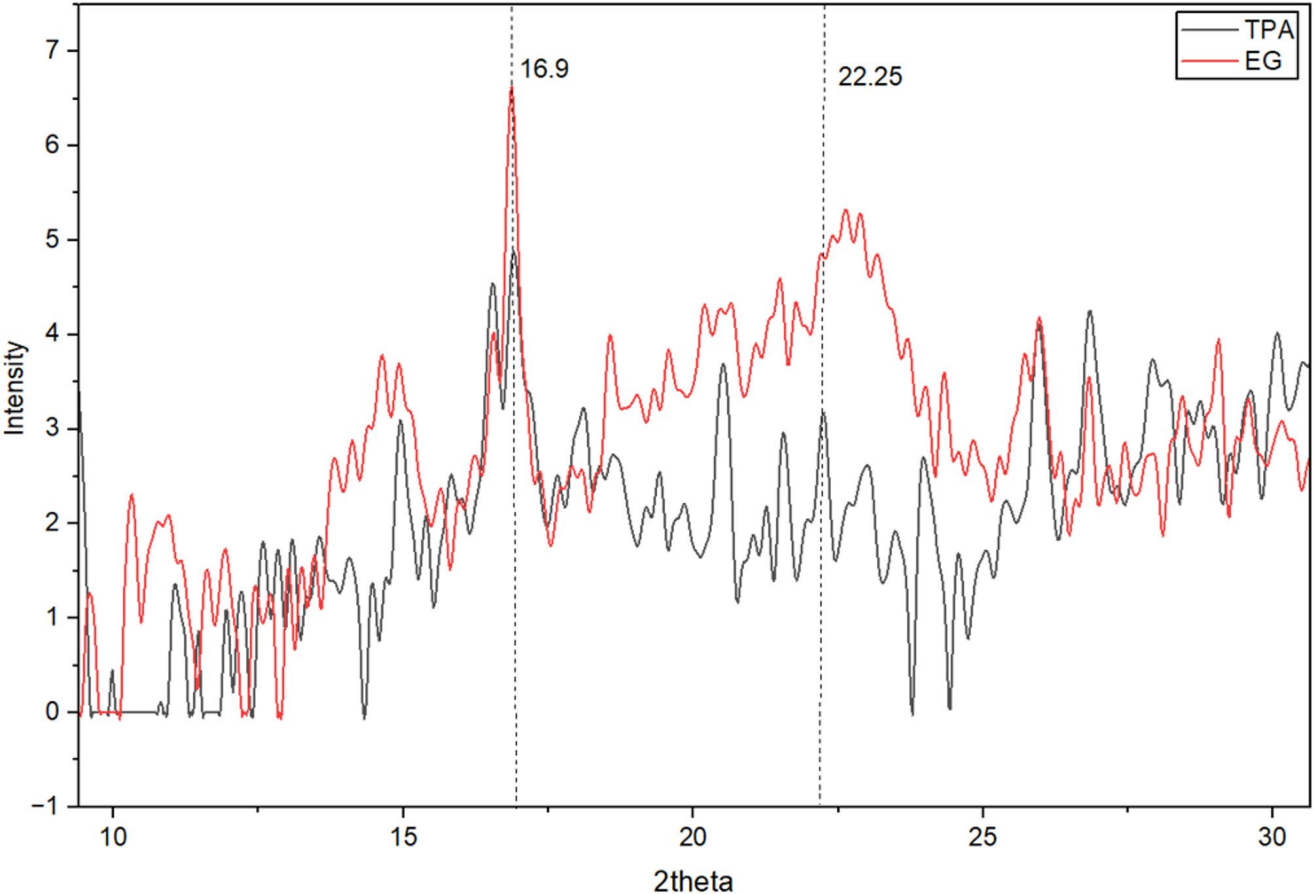
XRD spectra of TPA and EG with the highlighted angles used for the Segal approach highlighted by vertical dashed lines and an inset.

For samples obtained from growth on EG and TPA, X-ray diffraction (XRD) examination was used to assess the crystallinity index (CrI,%) of bacterial nanocellulose (BNC). The X-ray diffraction patterns observed in the 2θ range of 10–30° are shown in Fig. 3. The XRD patterns show cellulose type I’s distinctive reflections, indicating that the original crystalline structure has been preserved. Microcrystalline cellulose (cellulose Iα) is characterized by a diffraction peak at about 2θ = 16.9°, which corresponds to the (1–10)/(110) planes. Using the Segal approach, the crystallinity index was calculated using the prominent peak at 2θ = 22.25°, which represents the (200) crystallographic plane.

According to reports in the literature, BNC made from glucose usually has a CrI of about 70.5%, albeit this value is highly dependent on the carbon substrate and culture circumstances^38^. For instance, Abraham et al. found that nanocellulose made by *K. sucrofermentans* DSM 15,973 grown on glycerol had a higher crystallinity (82%). The CrI of 62.3% for BNC formed from EG in this investigation was lower than the usual value for BNC made from glucose. With a CrI of 18%, the TPA-derived BNC showed an additional significant drop in crystallinity. This significant decrease in crystalline ordering implies that different carbon substrates have an impact on cellulose production, which is reflected on microfibrillar packing and hydrogen bonding interactions. The lower CrI values observed for BNC cultivated in EG and TPA, compared to glucose-based systems, are consistent with the structural modifications indicated by FT-IR analysis, supporting the presence of increased amorphous character in these samples.

### Raman spectroscopy analysis of the BNC

All Raman spectra exhibited a low signal to noise ratio due to a strong fluorescent background, which was handled in the spectral Raman spectral preprocessing. To get the best preprocessing conditions Raman spectra from the pure carbon sources, nutrient buffers, and the microscope glass background were taken, see Fig. 3 and supplementary material S1. Note, this background was included in the background subtraction of the signal processing, but some of the peaks seen in Fig. 3 still had an influence on the analysis of the BNC Raman spectra that is discussed furtheron.

**Fig. 4:**
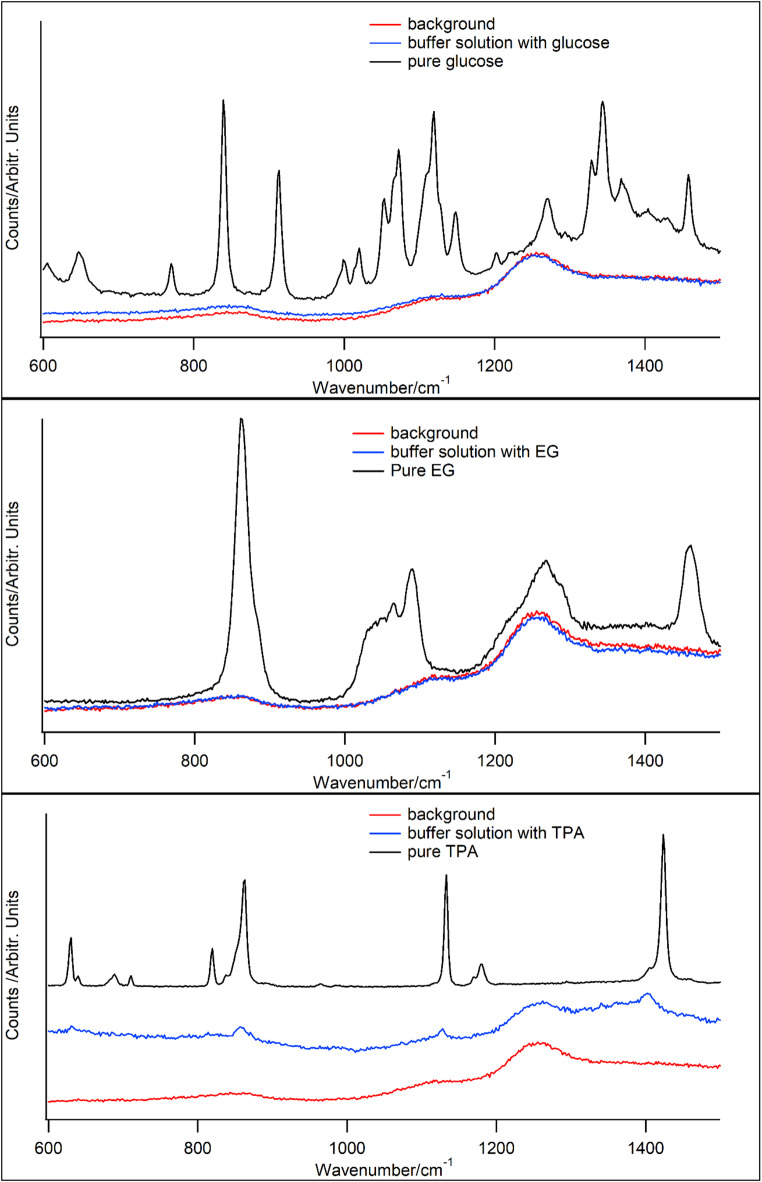
reveals that the dissolved feedstocks only give an impact on the background Raman spectrum for TPA. Basically, all sharp Raman bands of the pure feedstocks disappear.

In Fig. 4 Raw stacked Raman spectra of the pure substances (black line), buffer solutions with the carbon source (blue line), and microscope glass background (red line) for glucose (top), EG (middle), and TPA (bottom).

**Fig. 5:**
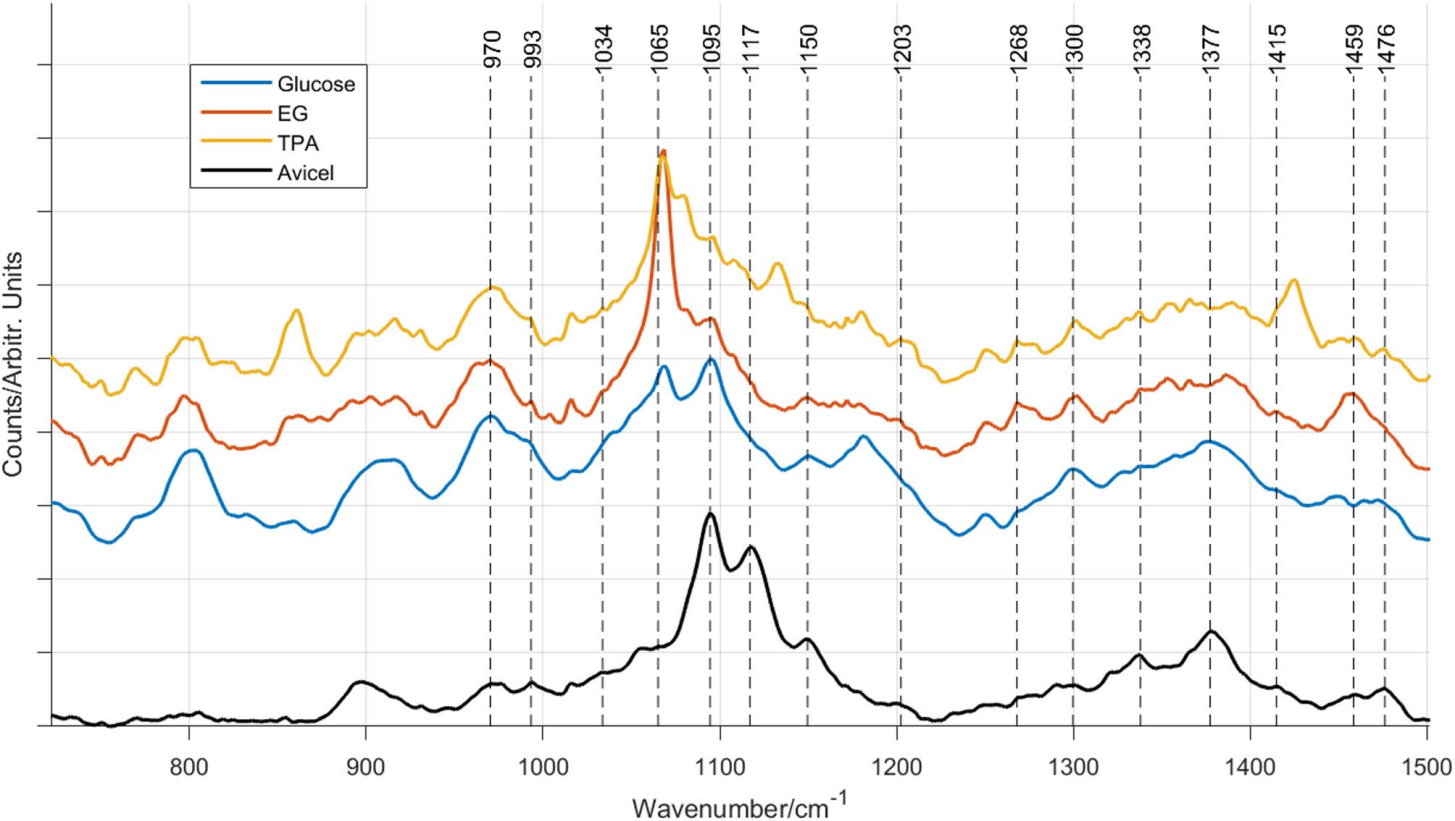
The normalized preprocessed Raman spectra for BNC fed with glucose (blue line), EG (red line), and TPA (yellow line) and for commercially produced cellulose from Avicel (black line). The dotted black horizontal lines mark the positions of the Raman bands.

Fig. 5 shows the preprocessed stacked Raman spectra of the different BNCs and the commercially available microcrystalline cellulose, Avicel. The black dotted vertical lines mark the assigned Raman bands listed in Table 1 according to references^25,39^. The crystallinity is traditionally ordered in terms of cellulose I, II or amorphous.

**Table 1:** Measured Raman bands for the different BNCs and Band assignemt from the litterature.

Measured Raman bands				Raman band^25,39^	Raman bands^25^		
Avicel	Glucose	EG	TPA	Assignments	Cellulose I	Cellulose II	Amorphous
			861 (s)	νPh 1 ring breath. (TPA)*			
970 (w)	970 (m)	970 (m)	968 (m)	Heavy atom (CC and CO): ρ(CH2)	971 (w)	969 (w)	970 (vw)
993 (w)	991 (w/m)	993 (w)	993 (w)	Stretch., C-C and C-O: ρ(CH2)	997 (w)	1000 (sh)	-
1034 (w/m)	1039 (sh/m)	1034/1039 (sh)	1034/1039 (sh)	Stretch., C-C and C-O: ν(CO) primary OH	1037 (sh)	-	-
	*1069 (s)*	*1067 (s)*	*1067 (s)*	*Shift Stretch.*,* C-C and C-O: ν(COC) glycosidic asym.*			
	*1094 (s)*	*1093 (s)*	*1096 (s)*	*Shift Stretch.*,* C-C and C-O: ν(COC) glycosidic asym.*			
1095 (s)				Stretch., C-C and C-O: ν(COC) glycosidic asym.	1096 (s)	1097 (s)	1092 (s)
1117 (m)	-	1119 (sh)	1117 (sh)	Stretch., C-C and C-O: ν(COC) in-plane sym.	1121 (s)	1117 (sh)	1116 (s)
			1135 (m)	νCX(X) (TPA)*			
1150 (m)	1150 (m)	1148 (w)	1146 (w)	Heavy atom stretch., HCC and HCO bend.: ν(CC)	1152 (s)	1146 (m)	-
1200 (w)	1206 (sh)	1201 (w)	1202 (w)	δ(COH): *δ*(CCH)	1201 (vw)	1197 (vw)	1199 (vw)
1268 (m)	1268 (sh)	1268 (m)	1268 (m)	HCC and HCO bend.: δ(CH2): *δ*(CH2) twisting	-	1265 (m)	1263 (m)
1299 (m)	1299 (m)	1299 (m)	1302 (m)	HCC and HCO bend.: δ(CH2) twisting	1294 (m)	-	-
1338 (m)	1338(m)	1340 (m)	1338 (m)	HCC, HCO, and HOC bend.: δ(CH2)	1339 (m)	1338 (m)	-
1377 (m/s)	1377 (m)	1387 (m)	1372/1390 (m)	HCC, HCO, and HOC bend.: δ(CH2)	1380 (m)	1374 (m)	1377 (s)
1415 (w)	1413 (sh)	1415 (w)		HCC, HCO, and HOC bend.: *δ*(CH2)	1409 (sh)	1413 (sh)	-
			1425	νPh (TPA)*			
1459 (w)	1463 (vw)	1457 (s)	1462 (w)	HCH and HOC bend.: δ(CH2) sciss.	1463 (sh)	1462 (m)	1463 (m)
1476 (w)	1472 (w)	1476 (sh)	1476 (w)	HCH and HOC bend.: δ(CH2) sciss.	1476 (m)	-	-

Many of the Raman bands in the Avicel Raman spectrum can be seen in the BNC Raman spectra, which confirms an overall cellulose structure, as seen in the SEM images of Fig. 2. Regarding the crystalline structures of the different cellulose types the Raman band ∼ 1268 cm^− 1^ is attributed to amorphous cellulose structures, while the Raman bands at 1300 cm^− 1^ and 1475 cm^− 1^ are seen for more crystalline structures. Fig. 5 shows that the band at 1268 cm^− 1^ is more pronounced for the BNC that had TPA and EG as a carbon source, while it is weaker when glucose was used as a feedstock, which indicates an amorphous structure for these BNCs. The band at 1300 cm^− 1^ and 1475 cm^− 1^ are equally strong in all BNC Raman spectra indicating that independently of the carbon sources there may be some crystalline structures, which is confirmed by the SEM images shown in Fig. 2. However, in the case of the TPA BNC Raman spectrum seen in yellow in Fig. 5 reveals a Raman band around 1440 cm^− 1^, which may be attributed to the TPA feedstock, see Fig. 4. This may indicate that, also in agreement SEM, TPA based BNC may be an amorphous composite with residues from the feedstock.

In the region between 700 and 900 cm^− 1^ and between 1170 and 1250 cm^− 1^, both the EG and TPA cultured BNC Raman spectrum show broad bands that cannot be connected to Ref^25^. Figure 3 shows that all carbon sources have a peak ∼ 800 cm^− 1^, which may indicate that not all carbon sources were valorized by the bacterium, however, the glass background had a broad hill at the same position, which may have remained, despite of careful preprocessing steps.

To investigate the spectral differences further, difference spectra between the commercially available cellulose (Avicel) and the BNC Raman spectra, were taken. Figure 5 shows the stacked difference Raman spectra between Avicel and the BNC spectra. Note that the solid black line marks the center line where the difference is zero in each spectrum.

**Fig. 6:**
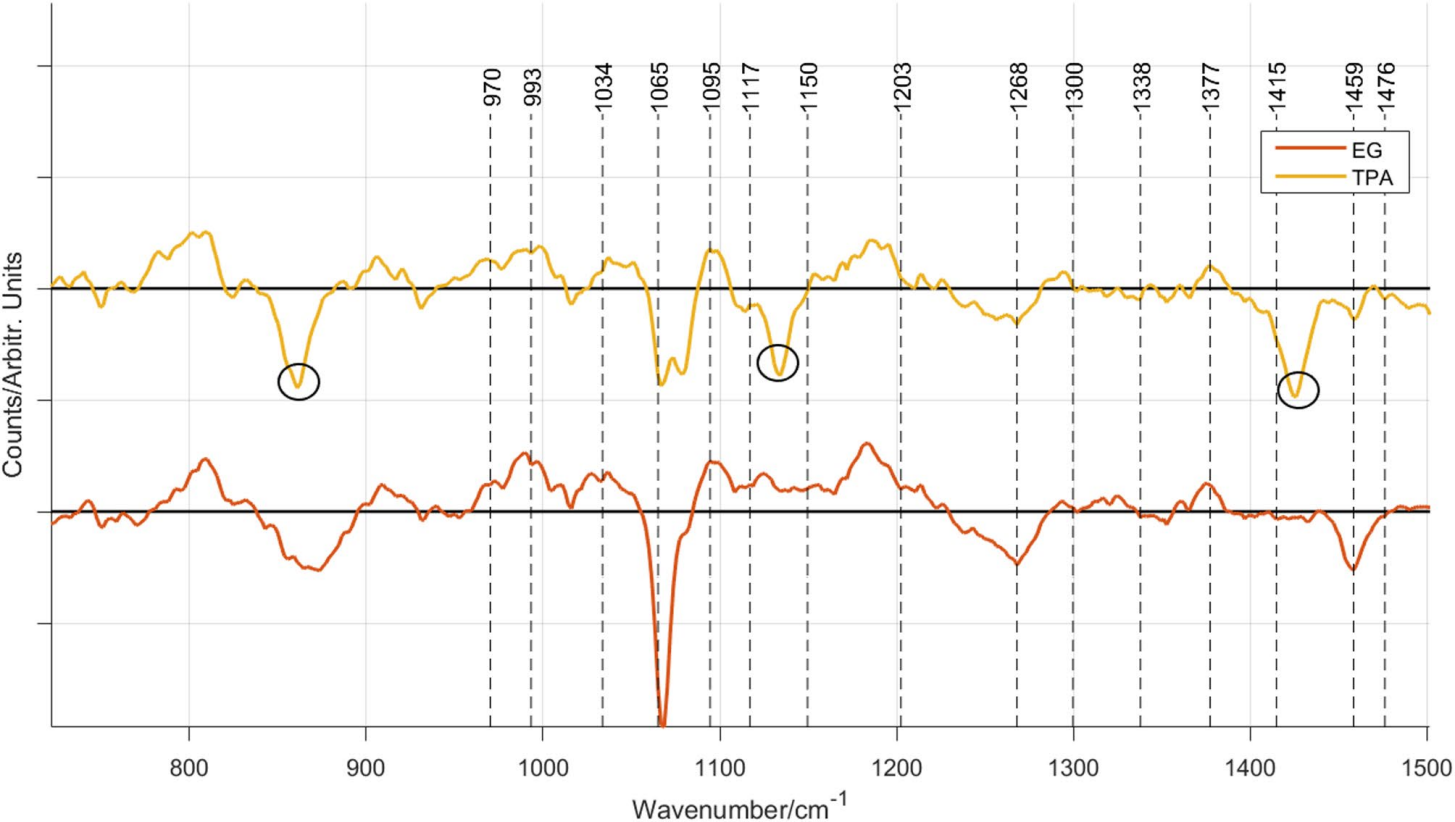
Difference spectra between the Avicel minus EG (red), and TPA (yellow). The dotted black lines mark the positions of Raman bands in Table 1. The black solid horizontal lines mark the zero line. The TPA spectrum’s circles around 861 cm^− 1^, 1135 cm^− 1^, and 1445 cm^− 1^ indicate leftovers from the carbon source.

The TPA-fed BNC Raman difference spectrum (yellow) shown in Fig. 6 shows typical TPA Raman bands, as identified in Table 1, namely at 861, 1135 cm^− 1^, and 1445 cm^− 1^. This indicated that *K. sucrofermentans* cannot valorize all TPA and that the resulting BNC may be a more amorphous composite. The broad hill around 800 cm^− 1^ is apparent in the BNC that had EG or TPA as a feedstock. The amorphous structures of these BNC were further confirmed looking at the band at 1268 cm^− 1^, which is below the zero line, see yellow and red spectrum in Fig. 6.

The biggest Raman spectral differences for all BNCs fed with different carbon sources can be seen in the region 1050–1117 cm^− 1^. The BNC Raman bands of the glycosidic side chain appear to be shifted to 1069 cm^− 1^ and 1095 cm^− 1^. In the Avicel Raman spectrum they are found at 1095 and 1117 cm^− 1^ according to^25^. It has earlier been reported^39^ that these Raman bands are sensitive to the orientation of the glycosidic linkage and the asymmetric breathing of the anhydro-glucose ring and can be shifted. Another study showed that the shift of these Raman bands towards lower wavenumbers can be caused by stress^40^. This needs further investigations.

### FT-IR spectroscopy analysis of bacterial nanocellulose (BNC)

**Fig. 7:**
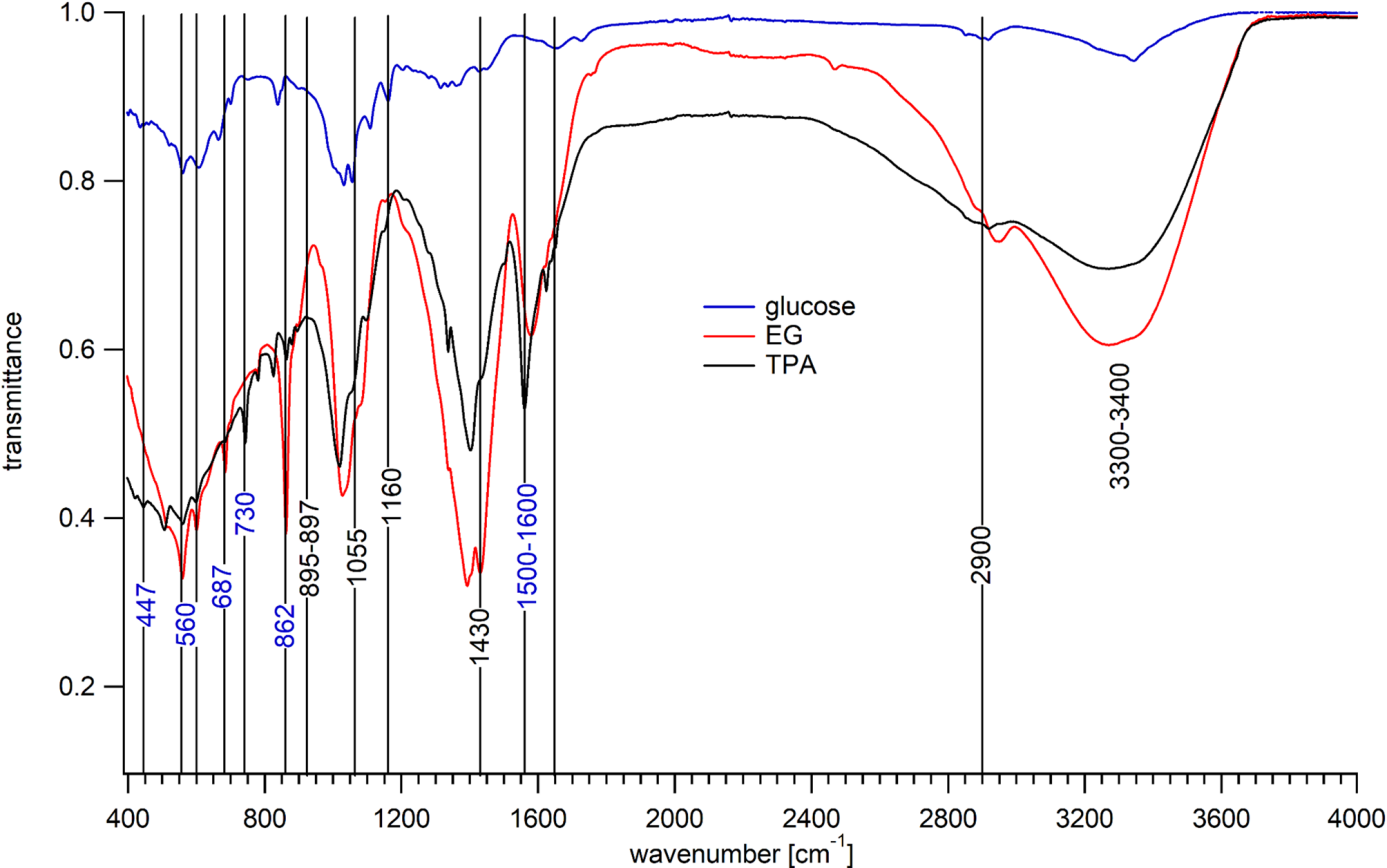
FT-IR spectrum for BNC produced by K. sucrofermentans cultivated in glucose, EG & TPA.

Fourier-Transform Infrared (FT-IR) spectroscopy was employed to characterize BNC from the three distinct carbon substrates (glucose, EG, and TPA) and to investigate the impact of the carbon source on the chemical structure and crystallinity of the resulting biopolymer. The untreated FT-IR spectra for all three BNC samples are presented in Fig. 7.

The overall spectral profiles seen in Fig. 7 show similar absorption bands, which indicates that the fundamental cellulose structure was synthesized in all cases, which is confirmed by the SEM images seen in Fig. 2. This finding confirms that *K. sucrofermentans* metabolized all three carbon sources, including the non-conventional substrates EG and TPA, to produce the target polymer. However, the transmission differed substantially between the feedstocks; glucose showed the highest transmission while it was substantially weaker for TPA and EG. This can be related to the density of the samples, which may indicate a heterogenous sample composition between the different BNCs in terms of brittleness and elasticity, which needs further investigations.

The characteristic absorption bands of cellulose were identified in accordance with standard interpretations^33–35,41^. They are highlighted by vertical lines in Fig. 7, note, lines with blue assignments belong to the carbon sources TPA and EG^42^. A broad and intense absorption band observed in the range of 3300–3400 cm⁻¹ for all samples is attributed to the O–H stretching vibration. This band is indicative of the extensive intra- and intermolecular hydrogen bonding network that is a hallmark of the cellulose structure and significantly influences its material properties^35^. The peaks observed at approximately 2900 cm⁻¹ correspond to C–H stretching vibrations from the methylene groups of the glucopyranose ring backbone. The band at ~ 1640 cm⁻¹, seen as a shoulder in TPA and EG, is associated with the H–O–H bending mode of adsorbed water, a common and expected feature in highly hydrophilic nanocellulose materials^23,35^. The most significant region for confirming the cellulose structure is the fingerprint region between 1200 and 1000 cm⁻¹. The strong, complex absorption bands here are assigned to C–O–C glycosidic ether linkage stretching (~ 1160 cm⁻¹) and C–O stretching vibrations within the pyranose ring (~ 1055 cm⁻¹)^32,33^. Furthermore, the presence of a characteristic peak at ~ 895 cm⁻¹, which is representative of the β-(1→4)-glycosidic linkages, provides a definitive fingerprint for cellulose and confirms the polymeric structure of glucose units in all BNC samples^43^ but especially for TPA and EG. The intensity and resolution of the absorption bands in the fingerprint region (1450–1000 cm⁻¹) are known to be sensitive to the degree of molecular order^34,35^. The relative crystallinity can be qualitatively assessed using the Lateral Order Index (LOI), often defined as the ratio of absorbance The A ~ 1430/A ~ 897 between the CH₂ symmetric bending, sensitive to crystallinity and the C–H deformation that is characteristic of amorphous regions^40^. The A ~ 1430/A ~ 897 ratio was calculated to 1.02 for glucose, 0.91 for TPA and 0.53 for EG, supporting the conclusion that the BNC produced with glucose as the feedstock possessed the most ordered crystalline. Further, pure TPA has a peak at 1425 cm⁻¹^43^ that may hamper the result of the ratio since it was not completely valorized, as confirmed by the Raman spectral data. The FT-IR spectra of this BNC exhibited the sharpest and most well-resolved peaks in this region, especially around 1055 cm⁻¹ and 1030 cm⁻¹. This is consistent with glucose being the natural and preferred metabolic precursor for cellulose synthesis, allowing for optimal enzymatic polymerization and crystallization^35^. In contrast, the spectra of BNC derived from EG and TPA showed broader and somewhat merged peaks, which suggests a higher proportion of amorphous content or a less ordered crystalline structure compared to the glucose-derived BNC^41^. This observation can be explained by the metabolic stress imposed on the bacteria when utilizing non-preferred carbon sources. The metabolic pathways required to convert EG and TPA into glucose precursors for cellulose polymerization are more complex and potentially less efficient, which may lead to irregularities in the chain extension and crystallization processes^43^. The lower crystallinity observed in BNC from alternative sources has been linked to altered physical properties, which can be a relevant factor for specific applications^35,41^. The variation in crystallinity based on the carbon source is a critical finding, as the crystalline-to-amorphous ratio directly impacts the mechanical strength, thermal stability, and water-holding capacity of nanocellulose materials^41^.

The purity of the synthesized BNCs was investigated by studying the non-cellulosic peaks. The FT-IR spectrum for TPA and EG derived BNC (Fig. 7) showed high characteristic aromatic C = C stretching bands (~ 1500–1600 cm⁻¹) and the carboxylic acid C = O stretch (~ 1690 cm⁻¹), as well as bands at 445, 530, 560, 687, 730, and 862 cm^− 1^ that typically are assigned to CO stretching and, respectively, to aromatic CH stretching of PET^42^. These bands indicate residual terephthalic acid monomers that were not completely incorporated into the polymer matrix^42,43^, revealing that not all of the EG and TPA were valorised by *K. sucrofermentans*. This is in good agreement with the Raman spectroscopic data for TPA, however, the Raman spectra of the BNC that had EG as a feedstock did not express the same clear remains of this carbon source, apart possibly from the Raman band ∼ 800 cm^− 1^.

## Conclusion

Molecular differences in BNC produced by *K. sucrofermentans* using glucose, EG, and TPA as a carbon source were investigated using Raman and FT-IR spectroscopy. The results were confirmed with gravimetric analysis, SEM and XRD. The best BNC yield was achieved when EG was used as a carbon source, while it was lowest for the TPA carbon source. The SEM images revealed that glucose as a feedstock produced BNC with the highest crystallinity, followed by EG and TPA. This finding could be verified by vibrational spectral data; the Raman bands identified in the microcrystalline plant cellulose Avicel could be identified in the BNC Raman spectra, all BNC showed crystallinity in accordance with the Raman band at 1300 cm^− 1^, revealing a crystalline structure, while the band at 1268 cm^− 1^ confirmed an amorphous structure for the BNC that were produced by EG and TPA as a carbon source, with TPA being slightly more crystalline. Interestingly, the BNC spectra revealed that the Raman bands in the 1050 cm^− 1^ − 1117 cm^− 1^ region were shifted to lower wavenumbers. These bands are sensitive to the orientation of the glycosidic linkage and the asymmetric breathing of the anhydro-glucose ring, and the shift may be caused by increased stress. This could not be confirmed by the FT-IR spectra. The TPA Raman spectrum showed additional bands at 629 cm^− 1^, 861 cm^− 1^, 1125 cm^− 1^, and 1425 cm^− 1^, while bands from the PET derived carbon sources appeared in the FT-IR spectra for both TPA and the EG. Furthermore, the crystallinity could be calculated using the absorption ratio A ~ 1430/A ~ 897, giving 1.02 for glucose, 0.91 for TPA and 0.53 for EG, supporting the conclusion that the BNC produced with glucose as feedstock for *K. sucrofermentans* possessed the most ordered crystalline structure, which was confirmed by the SEM images, XRD and partly by the Raman data. The different techniques showed complementary and strengthening results for the structural investigation of BNC, however, it has to be noted that the Raman spectral data required careful preprocessing, which was not necessary for the FT-IR spectral data. On the other hand, Raman spectroscopic data could indicate a strain on the glycosidic linkage, which was not possible with FTIR. To conclude, despite of that not all terephthalic acid monomers were valorised by *K. sucrofermentans*, the production of cellulose from plastic-derived monomers is a promising step towards sustainable material cycles and waste-cycling. Further work is needed to optimize the purification process to remove residual PET monomers from the final BNC membrane.

## Methods

### Microorganism and cultivation conditions

The study employed *K. sucrofermentans* (DSM 15973) purchased from DSMZ (the German Collection of Microorganisms and Cell Cultures). The bacterial strain was maintained as cryostocks in 15% (v/v) glycerol and stored at −80 °C. For inoculum preparation, 1 mL of cryopreserved culture was inoculated in the HS (Hestrin–Schramm) medium with the following composition (g/L): glucose-20; peptone-5; yeast extract-5; citric acid-1.15 and disodium hydrogen phosphate-2.7. The initial pH of the culture media was kept at pH = 6.8 and the culture was incubated in an orbital shaker at 28 °C and 150 rpm for 24 h^40^. For evaluating the potential of *K. sucrofermentans* to convert EG & TPA into BNC membranes, 1 L flasks containing 450 mL media with 20 g/L from three different carbon sources i.e., glucose, EG (VWR, Sweden) and TPA (disodium-terephthalic acid; TCI America) were inoculated with 10% (v/v) overnight grown culture and incubated statically at 28 °C for 21 days.

### Sample preparation

The synthesized BNC membrane was collected from the culture medium after 21 days. The BNC was alkali-treated with 1 M NaOH by boiling for 1 h to remove the adhered bacterial cells, followed by neutralization in boiling water for 2 h^43^. The wet BNC obtained was further lyophilized for 24 h to remove the water content and weighed gravimetrically. The yield refers to the amount (g) of dried BNC obtained per litre of culture.

### SEM settings

The characteristics of BNC produced under varying substrates were observed by scanning electron microscopy. The samples were placed on conductive carbon tape prior to the analysis, and the images were taken at a low accelerating voltage of 3 kV (FEI Magellan 400 XHR SEM, Everhart-Thornley detector).

### X-Ray diffraction analysis

XRD was employed to evaluate the crystallinity of BNC obtained from cultivation in EG and TPA. An X-ray diffractometer (PANalytical Empyrean XRD), which was fitted with a high-resolution Cu LFF HR X-ray tube and an advanced PIXcel3D detector, was employed. The X-ray diffraction patterns were recorded Ni-filtered Cu Kα radiation (λ = 1.54 A°). Diffractometer Type: Bruker D2 Phaser 2nd Gen. The generator current (mA) and operating voltage (kV) were 30 and 10; respectively. Data were collected at a rate of two degrees per minute between 5 and 80 degrees 2θ. Crystallinity degree of BNC was determined using the formula by Segal et al. : CrI(%) = ((I_002_ - I_am_)/I_002_)*100; where I_002_ is the intensity value for the crystalline cellulose, and I_am_ is the intensity value for the amorphous cellulose. The data was baseline corrected and smoothened using OriginLab.

### Raman spectroscopy settings

The Raman spectral experiments were performed using an Olympus IX73 inverted microscope (Olympus, Japan) coupled to a Shamrock 303i Raman spectrometer (Andor Technologies, UK). The Raman excitation laser source was a 785 nm 120 mW laser (Cobolt 08-NDL, Hübner Photonics, Ge). The power was reduced to 3–4 mW using a neutral density (ND) filter. The laser light was focused onto the sample using a 40x microscope objective (LUCPlanFLN, 40x/0.60, ∞/0–2/FN22, Olympus, Japan). The 180° backscattered Raman signal passed through a notch-, (785 nm StopLine single-notch filter, Semrock, USA) and an edge filter (785 nm EdgeBasic, Semrock, USA) to remove Raleigh scattered light. The remaining Raman scattered light was focused into an optical fiber guiding the signal into the spectrometer. The spectrometer was calibrated using the 1000 cm^− 1^ polystyrene Raman band. The exposure time was set to 1 s and 60 recordings were accumulated into one spectrum. 180 accumulated spectra were collected for each sample, resulting in an acquisition time of three hours. Further, Raman spectra of the microscope slide (background), three BNC samples, three pure carbon sources, three buffer solutions, and of the commercial nanocrystalline plant cellulose Avicel (PH-101, Merck Germany) were collected.

### Raman spectral data processing

The data was processed using MATLAB R2022b, (MathWorks, USA). The spectral window was set between 600 and 1500 cm^− 1^. The background and fluorescent signals were removed using an automated baseline fitting method^44^ based on Chebyshev polynomials with added smoothed and processed background spectrum, see Fig. S1. Finally, the first three Chebyshev polynomials of the first kind were used to fit the baseline for the glucose, EG, TPA and Avicel Raman spectra; see Fig. S2-S5 in supplementary material for more details. The final baseline was subtracted from all spectra. The cosmic rays were removed^45^ before calculating the mean spectra. The mean spectra were smoothed using the perfect smoother^46^ using uneven wavelength steps with the filter parameters d=1and λ = 10. Spectra were normalized by the normalize command with the zscore setting in MATLAB. The normalization caused parts of the spectra to have negative values, which was corrected by offsetting the spectra to a minimum value of zero.

### FT-IR spectroscopy settings

FT-IR spectra of the synthesized bacterial nanocellulose were registered with a Thermo Scientific™ Nicolet™ Summit FT-IR equipped with an Everest ATR. For analysis, the nanocellulose membranes were placed directly onto the ATR crystal, and spectra were recorded over a wavenumber range of 4000–650 cm⁻¹ at a spectral resolution of 2 cm^− 1^.

## Supplementary Information

Below is the link to the electronic supplementary material.

Supplementary Material 1

## Data Availability

All data generated or analysed during this study are included in this published article.
