# Supplementary material for: Characterization of bacterial nanocellulose cultivated on polyethylene terephthalate (PET) monomers via raman and fourier transform infrared spectroscopy
Source: Sci Rep. 2026 Apr 22;16:13133. doi: 10.1038/s41598-026-46886-z (PMC13103394; doi:10.1038/s41598-026-46886-z)
Supplement: Supplementary file 1 — Supplementary Material 1 [file 41598_2026_46886_MOESM1_ESM.docx]

# Supplementary material

### Fitting of background and Chebyshev polynomial to data

A polynomial can be fitted to data in the least square manner in MATLAB by solving the equation

$Ay=b$ (Eq. S1)

using the MATLAB command $y=A\backslash b$. $A$ is a $N\times M$ matrix, where each column contains one of the polynomial orders, $y$ is a $N\times1$ vector containing the fitting coefficients and $b$ is a $N\times1$ vector containing the measured data (i.e the mean spectra) to which a baseline is to be fitted to. A Chebyshev polynomial of the first kind was used in the fitting, and the recurrence formula are given by

$T_{0}\left( x \right)=1$ (Eq. S2 a)

$T_{1}\left( x \right)=x$ (Eq. S2 b)

$T_{n+1}\left( x \right)={2xT}_{n}\left( x \right)-T_{n-1}$ (Eq. S2 c)

where $T$ is a function of $x$ (wavenumber in this case). $n=1$ was used in the fitting of the Chebyshev polynomial, thus using the first three Chebyshev polynomial. The Chebyshev polynomial was structured into a matrix $C$ with the dimensions $N\times3$ as follows

$C= \left[ \begin{matrix} T_{0} & T_{1} & T_{2} \\ \vdots& \vdots& \vdots\\ T_{0} & T_{1} & T_{2} \end{matrix} \right]$. (Eq. S3)

The matrix $A$ was then constructed by adding a $N\times1$ vector $q$, which contained the processed background spectrum, to the Chebyshev matrix $C$ as follows:

$A=[\begin{matrix} C & q \end{matrix}]$. (Eq. S4)

The fitting was then performed as described in [33]. Note that before the background was included in the fitting, it was smoothed hard to remove noise (see Fig. S1), and then its mean value was subtracted, and finally, it was divided by its standard deviation.

### Figures S1-S5

In Fig. S1 the raw mean background spectrum and the smoothed background spectrum can be seen. The smoothed background was used to fit the baseline. In Fig. S2-S5 the raw mean spectra, blue line, and the fitted baseline, red line, for the glucose, EG, TPA and Avicel spectra can be seen respectively.


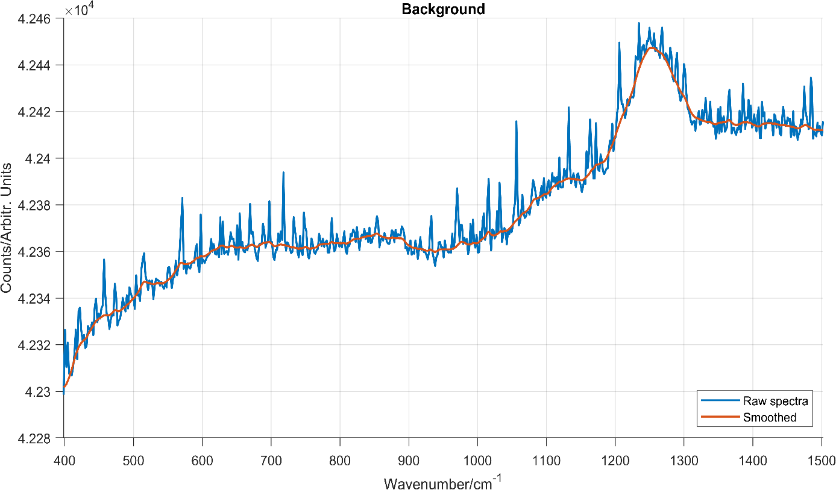


Fig. S1: The raw mean background spectrum, blue line, and the smoothed background spectrum, red line.


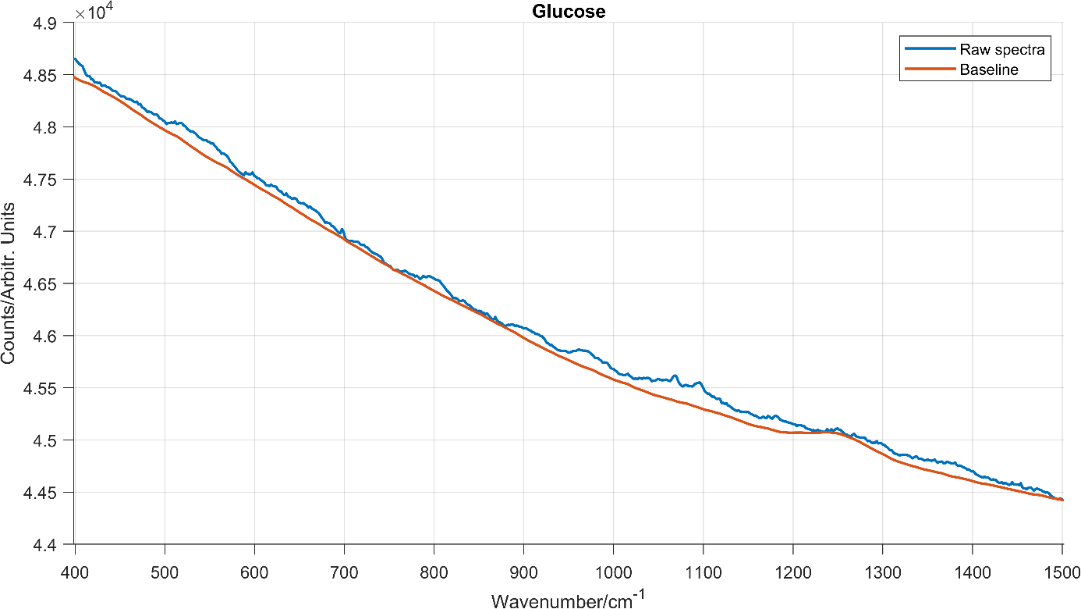


Fig. S2: The raw mean spectrum, blue line, for glucose BNC and the fitted baseline, red line.


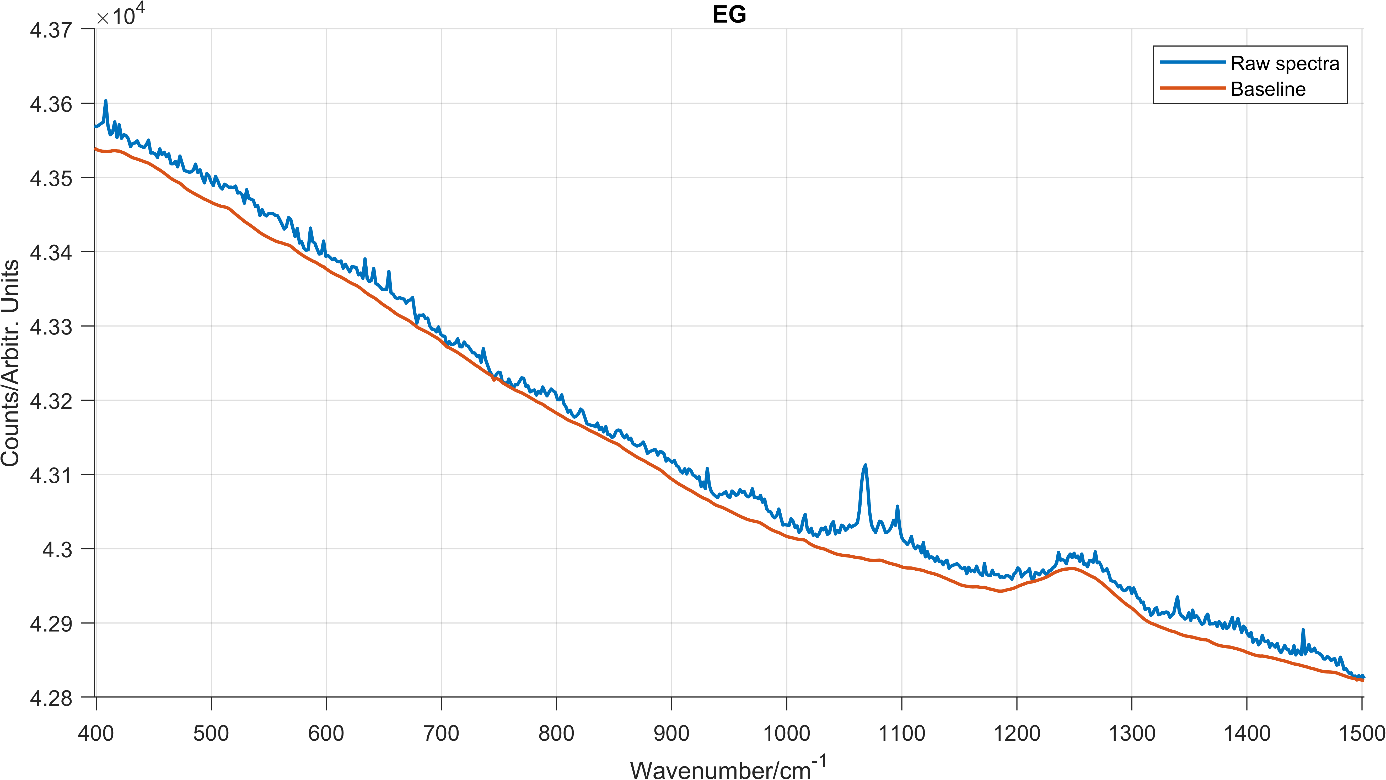


Fig. S3: The raw mean spectrum, blue line, for EG BNC and the fitted baseline, red line.


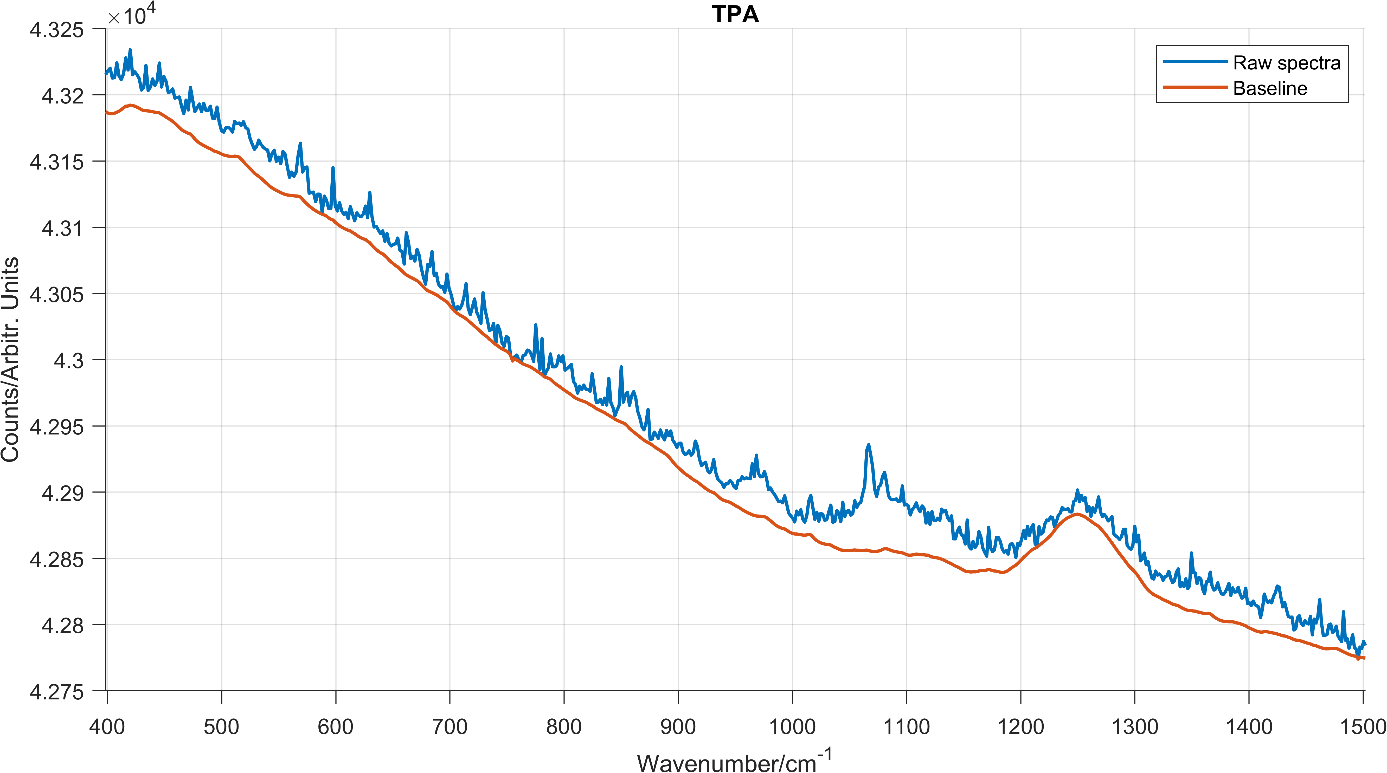


Fig. S4: The raw mean spectrum, blue line, for TPA BNC and the fitted baseline, red line.


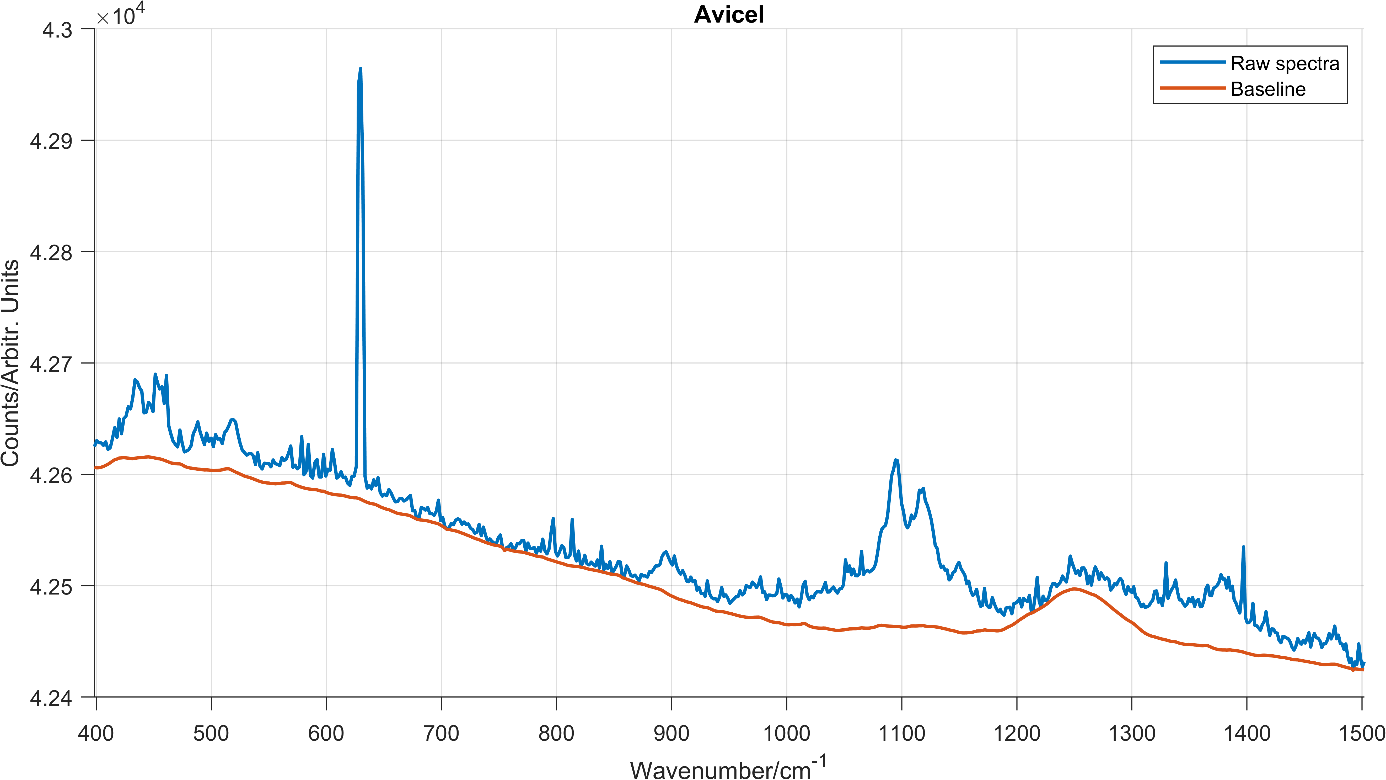


Fig. S5: The raw mean spectrum, blue line, for Avicel and the fitted baseline, red line.
